# Effect of oral or injectable supplementation with cobalamin in dogs with hypocobalaminemia caused by chronic enteropathy or exocrine pancreatic insufficiency

**DOI:** 10.1111/jvim.16528

**Published:** 2022-08-31

**Authors:** Chee‐Hoon Chang, Jonathan A. Lidbury, Jan S. Suchodolski, Joerg M. Steiner

**Affiliations:** ^1^ Department of Small Animal Clinical Sciences College of Veterinary Medicine & Biomedical Sciences, Texas A&M University College Station Texas USA

**Keywords:** alternative route, cobalamin deficiency, methylmalonic acid, small intestinal disease

## Abstract

**Background:**

Recent studies have shown similar efficacy of oral supplementation of cobalamin compared to injectable supplementation in dogs, but few prospective, randomized studies have been published.

**Objectives:**

To evaluate efficacy of oral or injectable supplementation with cobalamin in normalizing serum cobalamin and methylmalonic acid (MMA) concentrations in dogs with hypocobalaminemia caused by either chronic enteropathy (CE) or exocrine pancreatic insufficiency (EPI).

**Animals:**

Forty‐six client owned dogs with hypocobalaminemia.

**Methods:**

Prospective randomized clinical trial. Dogs were divided into 2 groups (CE or EPI), and randomized to receive oral or injectable supplementation of cobalamin. Each dog had 3 visits and serum cobalamin and MMA concentrations were measured at each visit.

**Results:**

In dogs with CE, serum cobalamin concentrations increased with oral (*P* = .02; median 149 [range 149‐231] to 733 [166‐1467] ng/L, median difference 552 [95% CI: 181‐899] ng/L) or injectable (*P* < .01; 168 [149‐233] to 563 [234‐965] ng/L, 367 [187‐623] ng/L) supplementation. In dogs with EPI, serum cobalamin concentrations increased with oral (*P* = .01; 162 [149‐214] to 919 [643‐3863] ng/L, 705 [503‐3356] ng/L) or injectable (*P* = .01; 177 [149‐217] to 390 [243‐907] ng/L, 192 [89‐361] ng/L) supplementation. Serum MMA concentrations decreased with oral or injectable supplementation in dogs with CE, but only with oral supplementation in dogs with EPI.

**Conclusions and Clinical Importance:**

Oral supplementation is an alternative for cobalamin supplementation in dogs with hypocobalaminemia caused by CE or EPI.

**Abbreviations**
BCS: body condition score
CE: chronic enteropathy
cPLI: canine pancreatic lipase immunoreactivity
cTLI: canine trypsin like immunoreactivity
EPI: exocrine pancreatic insufficiency
GI Lab: gastrointestinal laboratory
IF: intrinsic factor
MMA: methylmalonic acid
PERT: pancreatic enzyme replacement therapy
RI: reference interval
TVMDL: Texas A&M Veterinary Medical Diagnostic Laboratory

## 1 INTRODUCTION

Cobalamin is a required intracellular cofactor for 2 enzymes that are essential for maintaining cellular functions in mammals.^1^, ^2^, ^3^ Cobalamin that dogs obtain from their diet must combine with intrinsic factor (IF) before absorption in the ileum as a complex by specific receptors.^1^, ^4^ Because the exocrine pancreas is the main source for IF in dogs,^5^, ^6^ exocrine pancreatic insufficiency (EPI) is a common cause of hypocobalaminemia in dogs.^7^ Another common cause of hypocobalaminemia in dogs is chronic small intestinal disease where damaged ileal mucosa results in reduced cobalamin receptor expression with subsequently diminished cobalamin absorption.^8^ Other less common causes include congenital disorders of the cobalamin receptor and small intestinal dysbiosis.^1^, ^9^

Hypocobalaminemia in dogs can manifest as lethargy, inappetence, vomiting, diarrhea, weight loss, anemia, and failure to thrive in puppies.^1^, ^2^, ^10^ It is important that clinicians evaluate cobalamin status in dogs with chronic enteropathy (CE) or EPI because hypocobalaminemia is a negative prognostic factor in these diseases.^7^, ^8^ Serum cobalamin concentration is commonly measured to evaluate cobalamin status; however, it is not an accurate marker for cobalamin status on a cellular level. Methylmalonic acid (MMA) is a better marker for cellular cobalamin status because MMA accumulates when intracellular cobalamin level is abnormally low.^1^ Serum MMA concentration is a marker for cellular cobalamin status^11^, ^12^, ^13^; however, serum MMA concentration can also be affected by renal diseases or certain enzyme defects.^14^, ^15^ Both hypocobalaminemia and cobalamin deficiency are used interchangeably to describe low serum cobalamin concentrations. However, a serum cobalamin concentration below the lower limit of the reference interval (RI) should be referred to as hypocobalaminemia (≤250 ng/L) and hypocobalaminemia with an increased serum MMA concentration as cobalamin deficiency.^11^, ^13^

Many clinicians recommend supplementation regardless of the underlying etiology when serum cobalamin concentration is in the lower part of the RI (<400 ng/L at our institution).^16^ While subcutaneous administration of cobalamin is the mainstay of supplementation, recent oral supplementation is reported to be similarly efficacious in normalizing serum or cellular cobalamin concentration in dogs with CE or hereditary cobalamin malabsorption.^17^, ^18^, ^19^, ^20^ Among these studies, there are 2 prospective studies in dogs with CE evaluating the efficacy of oral supplementation of cobalamin.^18^, ^20^ Also, only 1 recent pilot study exists evaluating the efficacy of oral supplementation of cobalamin in dogs with EPI.^21^

The aim of this study was to evaluate the efficacy of oral or injectable supplementation with cobalamin in normalizing serum cobalamin and MMA concentrations in dogs with hypocobalaminemia caused by either CE or EPI. We hypothesized that both oral and injectable supplementation with cobalamin would have efficacy in normalizing serum cobalamin and MMA concentrations in dogs with hypocobalaminemia caused by CE or EPI.

## 2 MATERIALS AND METHODS

### 2.1 Animals

Client owned dogs were considered eligible for enrollment into this study based on the results of measurement of serum concentrations of cobalamin, folate, canine pancreatic lipase immunoreactivity (cPLI), and canine trypsin like immunoreactivity (cTLI) submitted to the Gastrointestinal Laboratory (GI Lab) at Texas A&M University. Veterinarians were instructed to collect all venous blood samples after withholding food for at least 10 to 12 hours. Inclusion criteria consisted of a serum cobalamin concentration below the lower limit of the RI (≤250 ng/L), a cPLI in the RI (≤200 μg/L), and a cTLI in the RI (5.7‐45.2 μg/L) or a cTLI below 2.5 μg/L, consistent with EPI. Dogs with any history of cobalamin supplementation or systemic, extragastrointestinal disease were excluded. Treatment of enrolled dogs beyond cobalamin supplementation was at the discretion of the attending veterinarian. Also, dogs could be fed any diet during the study period. Dogs were enrolled based on a history of signs of chronic GI disease for more than 3 weeks duration and results of blood work performed at baseline to exclude extra‐intestinal causes of the signs of GI disease. The blood work included CBC, serum chemistry profile, and the repeat GI panel (serum cobalamin, folate, cPLI, and cTLI). Signed informed consent was obtained from each owner before enrollment. The animal use protocol for this study was approved by the Animal Care and Use Committee of our institution (IACUC 2015‐0286, IACUC 2018‐0347).

### 2.2 Study design and cobalamin supplementation

This project was performed as a prospective randomized clinical trial. The enrolled dogs were classified into 2 different groups (CE or EPI) based on the cTLI results, with each dog then randomly assigned to 1 of 3 treatment groups (injectable supplementation of cobalamin, oral supplementation of cobalamin, or oral supplementation of cobalamin with folate), regardless of serum folate concentration at baseline. Randomization of each dog was achieved by following the ordered numbers prepared using the block randomization method performed by Nutramax Laboratories (Lancaster, South Carolina). Dogs in the injectable treatment group received weekly subcutaneous administration of cyanocobalamin (Vitamin B12 injection 1000 μg/mL, SPARHAWK Laboratories, Lenexa, Kansas) for 6 weeks, with an additional subcutaneous injection 4 weeks later for a total of 7 subcutaneous injections. Every subcutaneous injection was administered by a veterinary professional in a clinic setting. Dogs in the oral treatment groups (cobalamin only or cobalamin with folate) received daily oral supplementation using chews containing either cyanocobalamin or cyanocobalamin and folate, respectively, for a total of 12 weeks. Both chews were manufactured by Nutramax Laboratories (Lancaster, South Carolina). The dosage of cobalamin supplementation in each treatment group was determined based on the body weight of each dog (Table 1). Each dog had 3 visits with their primary veterinarian for baseline and 2 recheck visits during the study. During each visit, a pertinent history was obtained, a standard physical examination was performed, and a venous blood sample was drawn after withholding food for at least 10 to 12 hours. The first recheck was scheduled 6 weeks after initiation of cobalamin supplementation (week 7), and the second recheck was performed 1 week after cessation of supplementation (week 11 for injectable treatment group and week 13 for both oral treatment groups).

**TABLE 1:** Dosage of cyanocobalamin (±folate) supplementation for each dog in oral (per day) or injectable (per injection) treatment groups

Oral treatment	Body weight	<10 kg		10‐19 kg		≥20 kg		
	Dosage/day (cobalamin only)	250 μg (1 small chewable tablet)		500 μg (2 small chewable tablets)		1000 μg (1 large chewable tablet)		
	Dosage/day (cobalamin + folate)	250 + 50 μg (1 small chewable tablet)		500 + 100 μg (2 small chewable tablets)		1000 + 175 μg (1 large chewable tablet)		
Injectable treatment	Body weight	<10 lbs	10‐19 lbs	20‐39 lbs	40‐59 lbs	60‐79 lbs	80‐99 lbs	≥100 lbs
	Dosage/injection	250 μg	400 μg	600 μg	800 μg	1000 μg	1200 μg	1500 μg

### 2.3 Blood sample processing

After collection of a venous blood sample (about 5 mL), approximately 1 mL of whole blood was aliquoted into EDTA anticoagulant containing blood tubes, with the remaining whole blood was centrifuged to obtain serum. The separated serum (at least 1.5 mL) was aliquoted into plain red top tubes, and all samples were shipped to the GI Lab overnight on an icepack. When the blood samples were not shipped within the same business day, the blood tubes refrigerated (3‐5°C) overnight or over the weekend up to 48 hours. The EDTA containing blood tubes were submitted to the Texas A&M Veterinary Medical Diagnostic Laboratory (TVMDL) for a CBC. The serum samples were aliquoted into several sterile plastic tubes and evaluated for a chemistry profile, GI panel, and MMA concentrations.

### 2.4 Assays

For CBC, automated complete blood counts (ADVIA 120 Hematology System, Siemens, Erlangen, Germany) were performed and all blood smear slides were reviewed by a board‐certified clinical pathologist at the TVMDL. Chemistry profiles were automatically analyzed (AU 480 chemistry analyzer, Beckman Coulter, Indianapolis, Indiana) in the GI Lab. Serum cobalamin, folate, and cTLI assays were performed by automated competitive binding chemiluminescence immunoassay (IMMULITE 2000 XPi, Siemens, Erlangen, Germany) in the GI Lab. The RI for serum cobalamin in dogs at this laboratory is 251 to 908 ng/L, with lower and upper detection limits of 150 and 1000 ng/L, respectively. When the cobalamin concentration exceeded the upper detection limit, the serum sample was diluted and re‐run to obtain a numerical value. The laboratory's RI for serum folate and TLI in dogs are 7.7 to 24.4 and 5.5 to 45.2 μg/L, respectively. Serum MMA concentration was measured by the stable isotope dilution gas chromatography‐mass spectrometry method as described in previous studies,^11^, ^22^ and the RI for serum MMA in dogs was previously determined to 415 to 1193 nmol/L.^11^ Serum cPLI was measured using a commercially available ELISA kit (Spec cPL, IDEXX Laboratories, Westbrook, Maine) in the GI Lab, and the RI for cPLI in dogs is ≤200 μg/L at this laboratory.

### 2.5 Daily online questionnaire for owners

The owners of all enrolled dogs were asked to fill out a daily online questionnaire during the entire study period. The questionnaire consisted of several questions regarding ease of administration (for oral treatments only), appetite, vomiting, diarrhea, or any other adverse effects related to the cobalamin supplementation.

### 2.6 Statistical analysis

Serum cobalamin, folate, and MMA concentrations were statistically evaluated. Because none of the data were normally distributed, the Friedman tests followed by Dunn's multiple comparison tests were performed to compare the data (serum cobalamin, folate, and MMA concentrations) between different time points (baseline, first and second recheck) in each treatment group. Because no significant change of serum folate was identified in either oral treatment group (cobalamin only or cobalamin with folate), the data from both oral treatment groups were combined to increase statistical power. The final statistical analysis was performed using 2 treatment groups (combined oral group and injectable group) and shown in the results. Analysis was performed using a commercially available statistical software package (GraphPad Prism 9.2), and statistical significance was set at *P* < .05.

## 3 RESULTS

### 3.1 Animals

**FIGURE 1:**
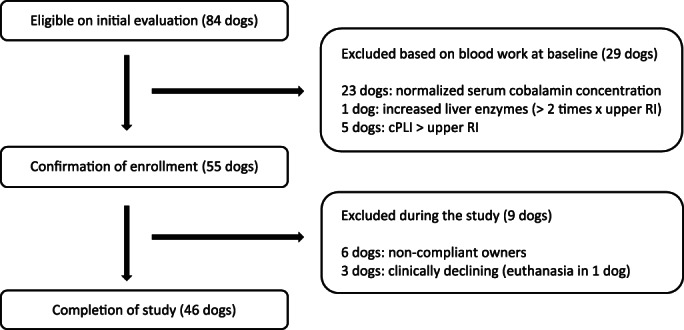
This flowchart shows the study process from initial evaluation to completion, including the number of dogs and reasons for exclusion at baseline and during the study

Eighty‐four client owned dogs were initially eligible for enrollment into the study. Among them, 46 dogs completed the study, and 38 dogs were excluded from the study because of various reasons as described in Figure 1. Among the remaining 46 dogs, 27 dogs were classified as having CE and 19 dogs as having EPI. Thirteen dogs with CE and 9 dogs with EPI received oral supplementation of cobalamin, and 14 dogs with CE and 10 dogs with EPI received injectable supplementation of cobalamin. Sixteen breeds of dogs with CE and 10 breeds of dogs with EPI completed the study (detailed information of the breeds is described in Data S1, Supporting Information). The median age of the CE and EPI group was 5 years old (range: 6 months‐12 years) and 4 years old (range: 1‐10 years), respectively. The sexual status of the CE group included 1 intact female, 9 spayed females, 3 intact males, and 14 neutered males. The sexual status of the EPI group consisted of 11 spayed females, 1 intact male, and 7 neutered males.

### 3.2 Relevant history and physical exam findings

Relevant history taking and standard physical examination were performed at 3 time points (ie, baseline, first and second rechecks) for each dog. In dogs with CE, the most common chief complaints at baseline were chronic diarrhea (21/27 dogs, 78%) and weight loss (9/27 dogs, 33%). Other complaints included intermittent vomiting, acute pica, inappetence, failure to gain weight, coprophagy, or occasional retching. Twenty‐four dogs with CE clinically improved over time with either oral or injectable supplementation of cobalamin, and 3 dogs (1 dog with oral and 2 dogs with injectable supplementation of cobalamin) had no clinical improvement. Fifteen dogs with CE (8 dogs with oral and 7 dogs with injectable supplementation) had other concurrent medical treatments other than cobalamin supplementation during the study, which included metronidazole (6 dogs), tylosin (3 dogs), fenbendazole (1 dog), anti‐inflammatory dose of prednisone (5 dogs), famotidine (1 dog), omeprazole (1 dog), cisapride (1 dog), probiotics (6 dogs), capromorelin (1 dog), or folate (3 dogs with injectable supplementation of cobalamin). No serious abnormalities were found on hysical examination in most dogs at baseline. The most common abnormality was a nonideal body condition score (BCS). Four dogs had a low BCS (2‐3/9) and 2 dogs had a high BCS (7‐8/9). Among 4 dogs with a low BCS, 2 dogs were supplemented orally with cobalamin and the other 2 dogs were treated with cobalamin by injection. The BCS of these dogs remained unchanged over time, despite clinical improvement with supplementation.

In dogs with EPI, the most common chief complaints at baseline were also chronic diarrhea (18/19 dogs, 95%) and weight loss (17/19 dogs, 90%), with 16 dogs (84%) being presented for both. All dogs with EPI were treated with pancreatic enzyme replacement therapy (PERT) and cobalamin supplementation (either oral or injectable), and all dogs showed clinical improvement over time. Two dogs with EPI had intermittently soft stool or diarrhea at the second recheck, which seemed most attributable to inconsistent PERT administration by the owner. Treatment protocol for most dogs with EPI consisted of PERT and cobalamin supplementations only, but 1 dog received concurrent oral administration of famotidine. No serious abnormalities were detected on physical examination at baseline, except for a low BCS (2‐3/9) in 9 dogs. All these 9 dogs gained weight over time (median 25.8 kg [3.8‐38.0 kg] at baseline and median 28.5 kg [4.2‐43.9 kg] at the second recheck), and their BCS became ideal (4‐5/9) at the second recheck.

### 3.3 Complete blood counts and chemistry profiles

Complete blood count and chemistry profiles were performed at 3 time points for each dog. The results are shown in Tables 2 and 3, and more detailed explanations are described in Data S1.

**TABLE 2:** Abnormalities of complete blood counts in dogs with chronic enteropathy (CE) or exocrine pancreatic insufficiency (EPI)

CE group (total 27 dogs)											
Parameter	High/low	Reference interval	Unit	Baseline (n = 27)				Second recheck (n = 26)			
				# dogs	% dogs	median	Results or range	# dogs	% dogs	Median	Results or range
Leukocytes	Leukopenia	6‐17	10^3^/μL	5	19	4.83	4.09‐5.29	5	19	5.05	4.12‐5.55
Eosinophils	Eosinophilia	0.1‐1.25	10^3^/μL	1	4	N/A	1.4	4	15	1.95	1.3‐3
Lymphocytes	Lymphopenia	1‐4.8	10^3^/μL	3	11	0.7	0.3‐0.9	6	23	0.7	0.5‐0.9
Neutrophils	Neutrophilia	3‐11.5	10^3^/μL	1	4	N/A	12.4	‐	‐	‐	‐
	Neutropenia	3‐11.5	10^3^/μL	3	11	2.8	2.2‐2.9	2	8	2.7	2.6‐2.8
Hematocrit (Hct)	Increased Hct	32‐55	%	4	15	58.3	55.9‐60.6	7	27	57	55.8‐63.3
Thrombocytes	Thrombocytosis	200‐500	10^3^/μL	4	15	671.5	601‐762	2	8	666	576‐756
	Thrombocytopenia	200‐500	10^3^/μL	1	4	N/A	186	1	4	N/A	171
	Thrombocytopenia with clumps	200‐500	10^3^/μL	3	11	134	97‐136	1	4	N/A	192

EPI group (total 19 dogs)											
Parameter	High/low	Reference interval	Unit	Baseline (n = 19)				Second recheck (n = 18)			
				# dogs	% dogs	Median	Results or range	# dogs	% dogs	Median	Results or range
Leukocytes	Leukocytosis	6‐17	10^3^/μL	1	5	N/A	17.85	‐	‐	‐	‐
	Leukopenia	6‐17	10^3^/μL	1	5	N/A	4.99	2	12	5.25	5.08‐5.42
Eosinophils	Eosinophilia	0.1‐1.25	10^3^/μL	2	11	1.75	1.7‐1.8	1	6	N/A	2.4
Lymphocytes	Lymphocytosis	1‐4.8	10^3^/μL	2	11	7.2	5.1‐9.3	2	12	7.85	7.3‐8.4
Neutrophils	Neutrophilia	3‐11.5	10^3^/μL	1	5	N/A	12.7	1	6	N/A	12.4
	Neutropenia	3‐11.5	10^3^/μL	1	5	N/A	2.6	‐	‐	‐	‐
Hematocrit (Hct)	Increased Hct	32‐55	%	1	5	N/A	56.6	4	24	56.7	55.6‐58.1
Thrombocytes	Thrombocytopenia	200‐500	10^3^/μL	1	5	N/A	105	1	6	N/A	127
	Thrombocytopenia with clumps	200‐500	10^3^/μL	5	26	110	86‐166	2	12	108	41‐175

Abbreviation: N/A, not applicable.

**TABLE 3:** Abnormalities of chemistry profiles in dogs with chronic enteropathy (CE) or exocrine pancreatic insufficiency (EPI)

CE group (total 27 dogs)											
Parameter	High/low	Reference interval	Unit	Baseline (n = 27)				2nd recheck (n = 26)			
				# dogs	% dogs	Median	Results or range	# dogs	% dogs	Median	Results or range
BUN	↑ BUN	8‐30	mg/dl	3	11	37	31‐43	1	4	N/A	31
	↓ BUN	8‐30	mg/dl	2	7	6.5	6‐7	‐	‐	‐	‐
Creatinine	↑ Creatinine	0.5‐1.4	mg/dl	2	7	1.85	1.7‐2	2	8	1.55	1.5‐1.6
Calcium	↓ Calcium	7.2‐12.8	mg/dl	1	4	N/A	7.1	1	4	N/A	6.3
Phosphorus	↑ Phosphorus	2.3‐6.5	mg/dl	2	7	8.45	7.4‐9.5	1	4	N/A	15.8
Albumin	↑ albumin	2.4‐4.3	g/dl	‐	‐	‐	‐	1	4	N/A	5.4
	↓ Albumin	2.4‐4.3	g/dl	5	19	1.5	1.4‐2.0	3	12	2	1.5‐2.2
Globulin	↑ Globulin	1.5‐4.5	g/dl	‐	‐	‐	‐	2	8	5.65	5.3‐6
	↓ Globulin	1.5‐4.5	g/dl	1	4	N/A	0.8	‐	‐	‐	‐
Total protein	↑ Total protein	5.6‐7.9	g/dl	‐	‐	‐	‐	1	4	N/A	11.5
	↓ Total protein	5.6‐7.9	g/dl	14	52	4.8	2.9‐5.4	12	46	4.8	2.7‐5.5
ALP	↑ ALP	12‐122	U/L	3	11	142	137‐202	3	12	248	153‐496
ALT	↑ ALT	13‐79	U/L	2	7	106	94‐118	6	23	111.5	96‐1265
GGT	↑ GGT	0‐25	U/L	1	4	N/A	27	‐	‐	‐	‐
Cholesterol	↓ Cholesterol	124‐335	mg/dl	5	19	103	37‐123	1	4	N/A	58.2

EPI group (total 19 dogs)											
Parameter	High/low	Reference interval	Unit	Baseline (n = 19)				2nd recheck (n = 18)			
				# dogs	% dogs	Median	Results or range	# dogs	% dogs	Median	Results or range
Glucose	↑ Glucose	60‐120	mg/dl	2	11	131	127‐135	‐	‐	‐	‐
BUN	↑ BUN	8‐30	mg/dl	1	5	N/A	34	‐	‐	‐	‐
Creatinine	↑ Creatinine	0.5‐1.4	mg/dl	‐	‐	‐	‐	1	6	N/A	1.5
Phosphorus	↑ Phosphorus	2.3‐6.5	mg/dl	‐	‐	‐	‐	1	6	N/A	6.9
Albumin	↓ Albumin	2.4‐4.3	g/dl	2	11	1.9	1.5‐2.3	‐	‐	‐	‐
Globulin	↑ Globulin	1.5‐4.5	g/dl	1	5	N/A	4.7	‐	‐	‐	‐
Total protein	↑ Total protein	5.6‐7.9	g/dl	‐	‐	‐	‐	2	11	8.9	8‐9.8
	↓ Total protein	5.6‐7.9	g/dl	4	21	5.3	5‐5.5	‐	‐	‐	‐
ALP	↑ ALP	12‐122	U/L	2	11	169.5	147‐192	1	6	N/A	132
ALT	↑ ALT	13‐79	U/L	4	21	113	92‐219	1	6	N/A	118
GGT	↑ GGT	0‐25	U/L	2	11	36.8	32‐41.6	‐	‐	‐	‐
Cholesterol	↑ Cholesterol	124‐335	mg/dl	‐	‐	‐	‐	2	11	375.5	366‐385
	↓ Cholesterol	124‐335	mg/dl	2	11	97	85‐109	‐	‐	‐	‐

Abbreviations: ALP, alkaline phosphatase; ALT, alanine transaminase; BUN, blood urea nitrogen; GGT, gamma‐glutamyl transferase; N/A, not applicable.

### 3.4 Serum cobalamin concentrations

**FIGURE 2:**
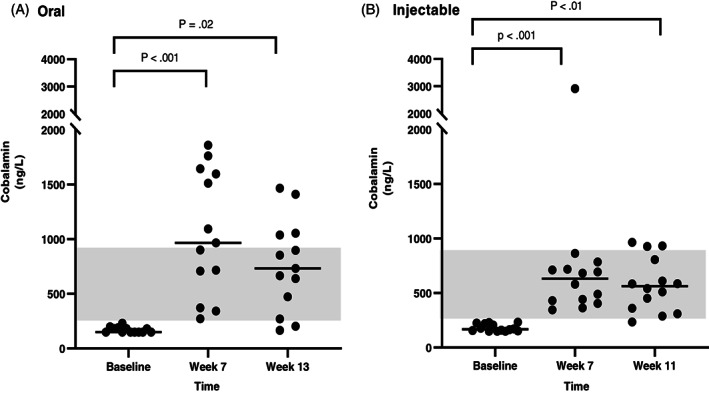
Serum cobalamin concentrations (ng/L) at different time points (baseline, first recheck, second recheck) in dogs with CE that received either oral (A, n = 13) or injectable (B, n = 14) supplementation with cobalamin. The RI for serum cobalamin concentration (251‐908 ng/L) is shown as a shaded gray area, and the bar shows the median of each dataset

**FIGURE 3:**
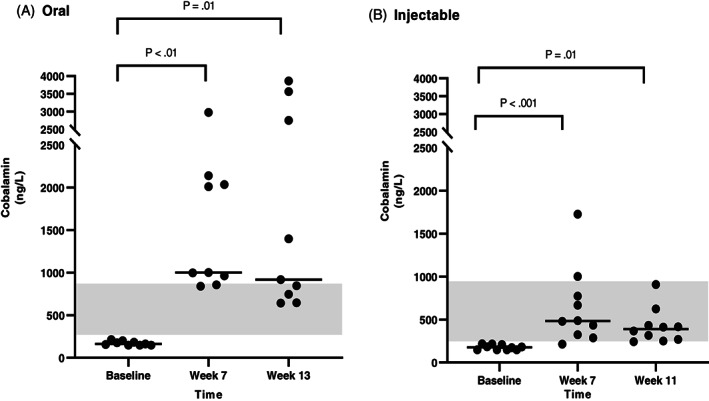
Serum cobalamin concentrations (ng/L) at different time points (baseline, first recheck, second recheck) in dogs with EPI that received either oral (A, n = 9) or injectable (B, n = 10) supplementation with cobalamin. The RI for serum cobalamin concentration (251‐908 ng/L) is shown as a shaded gray area, and the bar shows the median of each dataset

Serum cobalamin concentrations at 3 different time points were available for all dogs that completed the study. Results (median and range) of serum cobalamin and MMA concentrations at each time point, and also the difference (Δ) of serum cobalamin and MMA concentrations between each recheck and baseline are shown in Table 4. In the CE group, both oral and injectable supplementation of cobalamin induced a significant increase in serum cobalamin concentration between baseline and both rechecks (Figure 2). Among 13 dogs with CE that received oral supplementation of cobalamin, 3 dogs (23%) had a serum cobalamin concentration of less than 400 ng/L at both rechecks, suggesting the need for further supplementation. In dogs with CE receiving injectable supplementation of cobalamin, 2/14 dogs (14%) at the first recheck and 3/14 dogs (21%) at the second recheck had serum cobalamin concentrations of less than 400 ng/L. In the EPI group, the results were similar, showing a significant increase of serum cobalamin concentrations between baseline and both rechecks in both oral and injectable treatment groups (Figure 3). None of dogs with EPI that received oral supplementation of cobalamin had a serum cobalamin concentration less than 400 ng/L at either recheck. In contrast, among those dogs that received injectable supplementation of cobalamin, 3/10 dogs (30%) and 5/10 dogs (50%) at the first and second recheck, respectively, had a serum cobalamin concentration of less than 400 ng/L.

**TABLE 4:** Results (median and range) of serum cobalamin and methylmalonic acid concentrations at each time point in dogs with chronic enteropathy (CE) or exocrine pancreatic insufficiency (EPI) that received either oral or injectable cobalamin supplementation

Measurement (reference interval)	Patient group	Cobalamin supplementation	Baseline		First recheck				Second recheck			
			Median	Range	Median	Range	Δ median	Δ range	Median	Range	Δ median	Δ range
Cobalamin (251‐908 ng/L)	CE	Oral (n = 13)	149	149‐231	966	272‐1861	775	41‐1712	733	166‐1467	552	(−)65‐1318
		Injectable (n = 14)	168	149‐233	632	346‐2912	436.5	143‐2706	563	234–965	366.5	85‐767
	EPI	Oral (n = 9)	162	149‐214	1001	841‐2976	852	627‐2827	919	643‐3863	705	461‐3661
		Injectable (n = 10)	177	149‐217	483	214‐1726	288	65‐1577	390	243‐907	191.5	78‐758
Methylmalonic acid (415‐1193 nmol/L)	CE	Oral (n = 13)	1327	809‐4187	745	414‐1250	(−)670	171‐(−)3671	770	509‐1794	(−)814	462‐(−)3121
		Injectable (n = 14)	1127	601‐7757	826	566‐1955	(−)415.5	998‐(−)6461	725	518‐984	(−)390.5	276‐(−)7080
	EPI	Oral (n = 9)	1493	805‐3820	877	433‐2935	(−)468	1905‐(−)2898	723	536‐1278	(−)464	(−)82‐(−)3028
		Injectable (n = 10)	1376	584‐4921	1106	288‐2236	(−)373.5	990‐(−)3764	1132	505‐5418	(−)224.5	477‐(−)3794

*Note*: Δ indicates the difference between each recheck and baseline.

### 3.5 Serum folate concentrations

**FIGURE 4:**
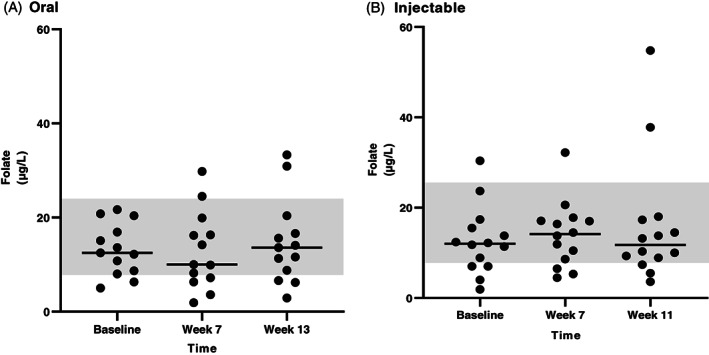
Serum folate concentrations (μg/L) at different time points (baseline, first recheck, second recheck) in dogs with CE that received either oral (A, n = 13) or injectable (B, n = 14) supplementation with cobalamin. The RI for serum folate concentration (7.7‐24.4 μg/L) is shown as a shaded gray area, and the bar shows the median of each dataset

**FIGURE 5:**
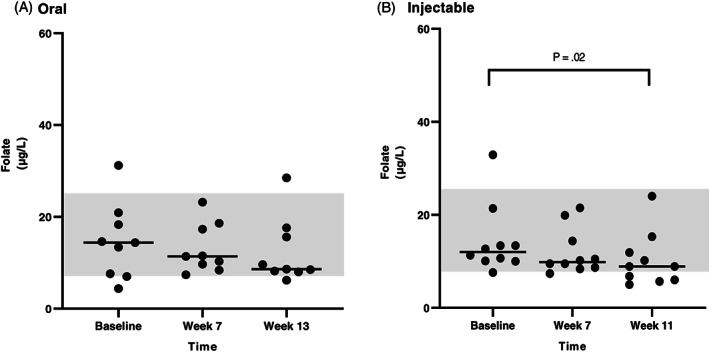
Serum folate concentrations (μg/L) at different time points (baseline, first recheck, second recheck) in dogs with EPI that received either oral (A, n = 9) or injectable (B, n = 10) supplementation with cobalamin. The RI for serum folate concentration (7.7‐24.4 μg/L) is shown as a shaded gray area, and the bar shows the median of each dataset

Serum folate concentrations were available at 3‐time points for all dogs that completed the study. The results are shown in Figures 4 and 5, and more detailed explanations are described in Data S1.

### 3.6 Serum MMA concentrations

**FIGURE 6:**
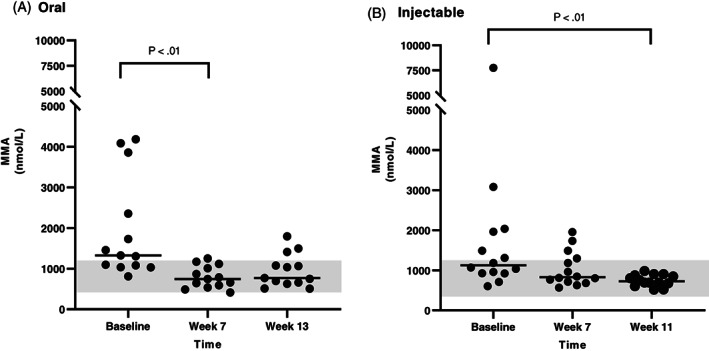
Serum MMA concentrations (nmol/L) at different time points (baseline, first recheck, second recheck) in dogs with CE that received either oral (A, n = 13) or injectable (B, n = 14) supplementation with cobalamin. The RI for serum MMA concentration (415‐1193 nmol/L) is shown as a shaded gray area, and the bar shows the median of each dataset

**FIGURE 7:**
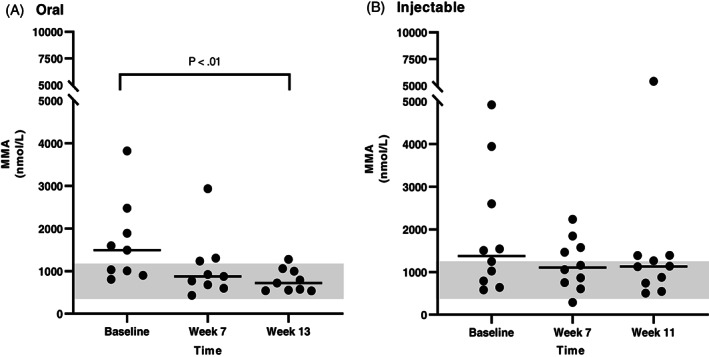
Serum MMA concentrations (nmol/L) at different time points (baseline, first recheck, second recheck) in dogs with EPI that received either oral (A, n = 9) or injectable (B, n = 10) supplementation with cobalamin. The RI for serum MMA concentration (415‐1193 nmol/L) is shown as a shaded gray area, and the bar shows the median of each dataset

**FIGURE 8:**
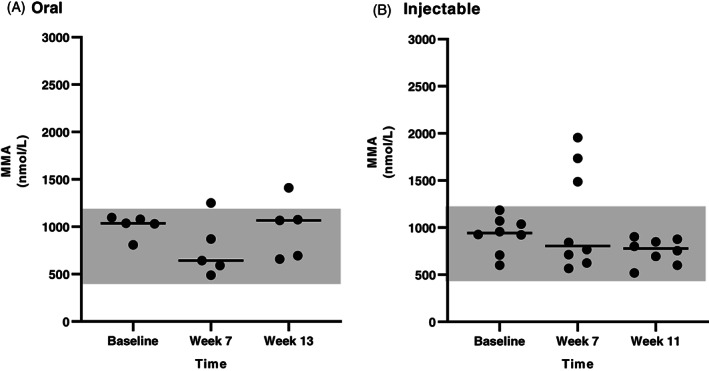
Serum MMA concentrations (nmol/L) at different time points (baseline, first recheck, second recheck) in dogs with CE that received either oral (A, n = 5) or injectable (B, n = 7) supplementation with cobalamin. In this figure, only dogs having serum MMA concentrations within the RI at baseline are included. The RI for serum MMA concentration (415‐1193 nmol/L) is shown as a shaded gray area, and the bar shows the median of each dataset

**FIGURE 9:**
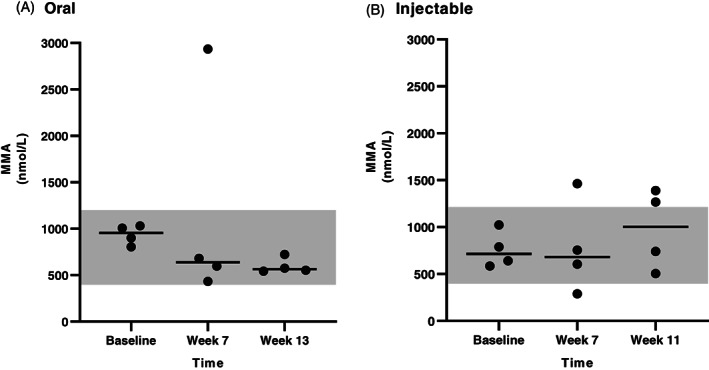
Serum MMA concentrations (nmol/L) at different time points (baseline, first recheck, second recheck) in dogs with EPI that received either oral (A, n = 4) or injectable (B, n = 4) supplementation with cobalamin. In this figure, only dogs having serum MMA concentrations within the RI at baseline are included. The RI for serum MMA concentration (415‐1193 nmol/L) is shown as a shaded gray area, and the bar shows the median of each dataset

Serum MMA concentrations were available for all dogs that completed the study for all 3 time points. In the CE group (Figure 6), serum MMA concentration was significantly decreased after either oral (between baseline and the first recheck) or injectable (between baseline and the second recheck) supplementation of cobalamin. In the EPI group (Figure 7), there was a significant decrease in serum MMA concentration between baseline and the second recheck in the oral treatment group, however, no significant difference in serum MMA concentration was identified between any time points in the injectable treatment group. When serum MMA concentrations were compared only in dogs having serum MMA concentration within the RI at baseline (13 dogs with CE and 8 dogs with EPI), no significant difference was identified over time with either oral or injectable supplementation of cobalamin for either CE or EPI dogs (Figures 8 and 9, respectively).

### 3.7 Serum cPLI and cTLI concentrations

In the CE group, all dogs in both the oral and the injectable treatment groups had normal cPLI and cTLI concentrations. In the EPI group, all dogs in either the oral or the injectable treatment groups had normal serum cPLI concentrations. Serum cTLI concentrations were consistent with EPI in all dogs of the EPI group.

### 3.8 Online daily questionnaire for owners

The results of the questionnaire are summarized in Table 5, and more detailed explanations are described in Data S1.

**TABLE 5:** Summary of daily online questionnaire responses in both treatment groups

		Oral treatment		Injectable treatment	
Dog group		CE (13 dogs)	EPI (9 dogs)	CE (14 dogs)	EPI (10 dogs)
Easiness of administration		13 (100%)	9 (100%)	N/A	N/A
Vomiting	1‐5 times total	3 (23%)	6 (66.6%)	5 (35.7%)	6 (60%)
	6‐10 times total	0	0	2 (14.3%)	0
	>10 times total	0	0	0	0
Diarrhea	1‐5 days total	3 (23%)	4 (44.4%)	8 (57.1%)	5 (50%)
	Intermittent	5 (38.5%)	1 (11.1%)	4 (28.6%)	1 (10%)
	Persistent	1 (7.7%)	0	0	0
Appetite	Same to increased	12 (92.3%)	9 (100%)	11 (78.6%)	9 (90%)
	Decreased to same	1 (7.7%)	0	3 (21.4%)	1 (10%)

## 4 DISCUSSION

The results of this prospective, randomized clinical trial indicate that oral or injectable supplementation of cobalamin is efficacious in normalizing serum cobalamin concentrations in dogs with hypocobalaminemia because of either CE or EPI, many of which had cobalamin deficiency as evidenced by an increased serum MMA concentration. Accordingly, both oral and injectable supplementation of cobalamin caused a significant decrease of serum MMA concentrations in dogs with CE; however, in dogs with EPI, only those supplemented with oral supplementation of cobalamin significantly decreased serum MMA concentrations.

The efficacy of oral cobalamin supplementation in normalizing serum cobalamin concentrations in dogs with various causes of hypocobalaminemia, including dogs with CE or EPI, is reported.^18^, ^19^ These studies were performed based on the results of human studies showing a similar efficacy of oral and injectable supplementation of cobalamin in normalizing serum cobalamin concentrations.^23^, ^24^, ^25^, ^26^ The first study in dogs was a retrospective study evaluating the efficacy of oral supplementation of cyanocobalamin in dogs with CE and low serum cobalamin concentration (≤270 ng/L, RI: 234‐811 ng/L).^19^ There was a significant increase of serum cobalamin concentration with all dogs achieving a serum cobalamin concentration within the RI after treatment. Important study limitations were that quantification of serum MMA was not performed as well as incomplete differentiation between dogs with CE from those with EPI. A lack of standardized time points for rechecking cobalamin status after initiation of supplementation resulted in substantial variation for recheck intervals (20‐202 days). A prospective study comparing the efficacy of orally administered cyanocobalamin versus subcutaneously administered hydroxocobalamin supplementation in dogs with CE and a decreased serum cobalamin concentration (<285 ng/L, RI: 244‐959 ng/L) demonstrated a significant increase of serum cobalamin concentrations after oral supplementation of cyanocobalamin.^18^ This study did not include dogs with EPI, so the efficacy of oral supplementation in dogs with EPI was not evaluated. Also, serum MMA concentrations evaluating the cellular cobalamin status were not reported in this study; however, they were evaluated in the same group of dogs and reported separately.^20^ Results of this study showed a significant decrease of serum MMA concentrations with both oral and injectable supplementation of cobalamin.

A recent retrospective “pilot” study evaluated the effect of oral cyanocobalamin supplementation in dogs with EPI that had a low serum cobalamin concentration (≤350 ng/L, RI: 244‐959 ng/L).^21^ There was a significant increase of serum cobalamin concentrations after treatment suggesting that oral supplementation of cobalamin is a potential alternative to injectable treatment in dogs with EPI. Limitations of this study were the retrospective design and a lack of serum MMA concentrations for most dogs (ie, serum MMA was only available for 4 dogs).

Our results in dogs with CE identified that the median and median Δ change in cobalamin concentrations in oral treatment group were consistently higher than those of dogs in the injectable treatment group at both rechecks. These results are different from the previous prospective study in dogs with CE, showing higher mean cobalamin concentrations in injectable treatment group at the first recheck (Day 28) and in oral treatment group at the second recheck (Day 90).^18^ This difference might be because of the use of different types of injectable supplement (cyanocobalamin vs. hydroxocobalamin)^27^, ^28^, ^29^ or variations in the recheck times between the 2 studies. In a human study comparing the efficacy of a 90‐day course of either oral or intramuscular supplementation of cyanocobalamin, intramuscular supplementation caused consistently higher serum cobalamin concentrations than oral supplementation.^24^ Our study is the first prospective study to examine the efficacy of oral supplementation of cyanocobalamin in dogs with EPI. Our results are consistent with conclusions from the retrospective pilot study showing a significant increase of serum cobalamin concentrations after oral supplementation of cyanocobalamin in dogs with EPI.^21^ This finding is especially clinically relevant because hypocobalaminemia, a common complication in dogs with EPI, is the only independent risk factor associated with reduced survival in dogs with EPI, and cobalamin supplementation is recommended as a treatment regimen in dogs with EPI when a low serum cobalamin concentration is identified.^7^, ^30^ Having the option of oral supplementation of cobalamin might also be beneficial for clients in terms of the convenience of avoiding a weekly injections, considering most dogs with EPI are receiving pancreatic enzyme replacement therapy orally. Our study identified that the median and median Δ change in cobalamin concentrations in oral treatment group were consistently higher than in the injectable treatment group for both rechecks in dogs with EPI.

While the majority of dogs in this study had a significant increase in serum cobalamin concentration compared to baseline after either oral or injectable supplementation of cobalamin, some dogs showed little improvement in their serum cobalamin concentrations. In dogs with CE, both oral and injectable supplementation of cobalamin resulted in a similar percentage (<25%) of dogs that still had a serum cobalamin concentration of less than 400 ng/L after completion of the treatment protocol, necessitating further supplementation. This result is different from the previous study in dogs with CE, where no dogs receiving oral supplementation had serum cobalamin concentrations less than 400 ng/L at both rechecks.^18^ The result of our study is also dissimilar to previous studies in dogs and humans in which there was a gradual increase of cobalamin concentrations.^18^, ^23^, ^26^ This difference might be because of different time points for the second recheck, use of different cobalamin formulations, or inconsistent cobalamin administration by owners. Of the dogs with EPI, none had a serum cobalamin concentration of less than 400 ng/L after completion of oral supplementation, but 3 dogs (30%) at the first recheck and 5 dogs (50%) at the second recheck receiving subcutaneously administered cobalamin had serum cobalamin concentrations of less than 400 ng/L. This discrepancy in dogs with CE and EPI might be related to differences in the status of ileal cobalamin receptors in dogs with the 2 distinct diseases. These results also reiterate the importance of reassessing cobalamin status after completion of the currently recommended treatment protocol as not every dog achieved normalization of serum cobalamin concentrations.

Even though our study and other studies have demonstrated efficacy of oral supplementation of cobalamin in dogs with hypocobalaminemia caused by CE or EPI,^18^, ^19^, ^21^ the mechanism of orally administered cobalamin absorption in dogs with a compromised GI tract or in the absence of IF in dogs with EPI remains unknown.

In our study, serum MMA concentrations were measured in all dogs at all 3 time points to evaluate cobalamin status on a cellular level. In dogs with CE, both oral and injectable supplementation of cobalamin significantly decreased serum MMA concentrations, indicating an improved or normalized cobalamin status on a cellular level. This result is consistent with previous findings, showing a significant decrease of serum MMA concentrations with both oral and injectable supplementation of cobalamin in dogs with CE.^20^ In that study, there was a significant decrease of serum MMA concentration between baseline and the first recheck (Day 28) in both treatment groups and no significant change between the first recheck (Day 28) and the second recheck (Day 90). In our study, a significant decrease in MMA concentration was identified between baseline and the first recheck in the oral treatment group, and between baseline and the second recheck in the injectable treatment group. This difference between 2 studies might be because of use of a different formulation of injectable supplement (cyanocobalamin vs. hydroxocobalamin).

Only oral supplementation of cobalamin significantly decreased serum MMA concentrations in dogs with EPI despite both oral and injectable formulations resulting in a significant increase of serum cobalamin concentrations. This finding might be relevant because 30% to 50% of dogs with EPI that received subcutaneously administered cobalamin supplementation showed little improvement in their serum cobalamin concentration after completion of their treatment protocol. Also, none of the dogs with EPI orally supplemented with cobalamin had a serum cobalamin concentration less than 400 ng/L, indicating satisfactory responses to cobalamin supplementation in all dogs. One dog in the injectable group had a surprisingly higher MMA concentration (5418 nmol/L) at the second recheck compared to both baseline (2599 nmol/L) and the first recheck (1574 nmol/L), and the value at the second recheck (5418 nmol/L) was markedly higher compared to MMA concentrations in the remainder of the dogs (505‐1389 nmol/L) for that same time point (second recheck). To evaluate if the 1 outlier of the MMA concentration at the second recheck would change the results during the statistical analysis, the analysis was repeated after changing the MMA concentration at the second recheck to the same value as was measured during the first recheck (1574 nmol/L), however, the results of statistical analysis did not change. These dissimilar responses in MMA concentrations in addition to cobalamin status between dogs with CE and EPI might be related to different status of cobalamin receptors in the ileum or targeted cells or of transcobalamin in blood that deliver cobalamin to targeted cells. However, further studies evaluating larger numbers of dogs are needed to find out if these observed differences were related to the route of cobalamin application.

In our study, no dogs having a low normal serum cobalamin concentration at baseline were enrolled, which differed from previous studies.^18^, ^19^, ^20^, ^21^ This strict inclusion criterion was implemented to ensure that only dogs likely to have cobalamin deficiency would be enrolled into the study. Even with this strict criterion, only 14/27 dogs (52%) with CE and 11/19 dogs (58%) with EPI had an increased serum MMA concentration at baseline indicative of cobalamin deficiency. All remaining dogs had serum MMA concentrations within the RI in the face of a severely decreased serum cobalamin concentration. In aforementioned studies, not all dog with hypocobalaminemia had increased serum MMA concentrations and the percentage of dogs with an increased MMA concentration at baseline varied slightly more than in our population (25%‐46%).^11^, ^16^, ^20^ Our finding of a higher percentage of dogs with an increased baseline serum MMA concentration compared to the previous studies might be explained by the more stringent enrollment criteria. While the exact mechanism responsible for a normal serum MMA concentration despite a severely low serum cobalamin concentration is unknown, it might be that these dogs are earlier in their course of disease, preceding depletion of intracellular cobalamin stores.

Results of the online owner questionnaire showed that clinical complications of both oral and injectable treatment were rare and both routes seemed to be well tolerated by the dogs enrolled in this study. More detailed explanations about online questionnaire data are described in the supplemental file. Among the excluded 38 dogs, 23 dogs were excluded before a normalized serum cobalamin concentration at baseline even though they had cobalamin concentrations lower than the lower limit of the RI upon initial evaluation for eligibility. The more detailed explanations about this finding are described in Data S1.

This study had several limitations. First, classification of dogs as CE was based on the history and results of standard blood work. The majority of dogs did not have a full diagnostic work up including fecal examination and baseline cortisol concentration, abdominal ultrasound, and GI endoscopy to determine an underlying cause for the hypocobalaminemia beyond those with a diagnosis of EPI. Second, because diet was not controlled for differences in the cobalamin content of various diets consumed by enrolled dogs, it might have affected serum cobalamin concentrations. It is difficult to evaluate the exact impact of dietary cobalamin because dietary cobalamin has not been shown to have a consistent effect on serum cobalamin concentrations.^31^, ^32^ Third, each dog enrolled into this study received different therapies for their underlying diseases (CE or EPI) by their primary veterinarians, and these therapies might have influenced the level of disease control and consequently cobalamin concentrations even though no such influence was observed in a previous study.^19^ Fourth, among many different breeds of dogs enrolled into our study, the Border Collie (3 dogs with CE and 1 dog with EPI) was the only breed that has been reported to be affected by hereditary cobalamin malabsorption. It would have been ideal if we had performed genetic testing on these Border Collies to exclude hereditary cobalamin malabsorption. However, considering the age of enrolled Border Collies (2‐6 years), congenital cobalamin malabsorption is less likely a cause of hypocobalaminemia in these dogs because the age of 5 Border Collies with congenital cobalamin malabsorption in 2 published case reports was 8 to 12 months with 1 additional dog being 41 months. Finally, we did not count the remaining number of chews at the end of each treatment period, which might have been helpful in excluding dogs of noncompliant owners.

## CONFLICT OF INTEREST DECLARATION

All authors are employed by the Gastrointestinal Laboratory at Texas A&M University, which offers measurement of serum cobalamin and methylmalonic acid concentrations on a fee for service basis. Dr Steiner acts as a consultant for Nutramax Laboratories. Dr Steiner and Dr Suchodolski also act as paid speakers for Nutramax Laboratories.

## OFF‐LABEL ANTIMICROBIAL DECLARATION

Authors declare no off‐label use of antimicrobials.

## INSTITUTIONAL ANIMAL CARE AND USE COMMITTEE (IACUC) OR OTHER APPROVAL DECLARATION

The animal use protocol for this study was approved by the Animal Care and Use Committee of Texas A&M University (IACUC 2015‐0286, IACUC 2018‐0347).

## HUMAN ETHICS APPROVAL DECLARATION

Authors declare human ethics approval was not needed for this study.

## Supporting information


**Data S1** Supporting Information.
