# Supplementary material for: Effect of oral or injectable supplementation with cobalamin in dogs with hypocobalaminemia caused by chronic enteropathy or exocrine pancreatic insufficiency
Source: J Vet Intern Med. 2022 Aug 31;36(5):1607–21. doi: 10.1111/jvim.16528 (PMC9511088; doi:10.1111/jvim.16528)
Supplement: Supplementary file 1 — Data S1 Supporting Information. [file JVIM-36-1607-s001.pdf]

Supplementary information

Result

Animals

Sixteen different breeds of dogs with CE that completed the study included German shepherds (n=5), mixed breed dogs (n=5), Border collies (n=3), Labrador retrievers (n=2), and one dog for each of the following breeds: Australian cattle dog, Bernese mountain dog, Rottweiler, Boston terrier, Boxer, Coon hound, Doberman pinscher, French bulldog, Irish setter, Italian greyhound, Shiloh shepherd, and Staffordshire terrier. Ten different breeds of dogs with EPI that completed the study included German shepherds (n=7), mixed breed dogs (n=3), Cavalier King Charles spaniels (n=2), and one dog for each of the following breeds: Akita, Border collie, Chihuahua, Golden doodle, Shih tzu, West Highland white terrier, and Yorkshire terrier.

Complete blood counts and chemistry profiles

Complete blood count and chemistry profiles were performed at three time points for each dog. However, 3 CBC data from 3 dogs (1 dog with CE at the first recheck and 2 dogs each with EPI, one at the first and at the second recheck) were unavailable due to hemolyzed blood samples. Also, 3 chemistry profile results from 3 dogs (2 dogs each with CE, one at the first and at the second recheck, and 1 dog with EPI at the second recheck) were not available due to an inadequate amount of serum. Consequently, 135 CBC and chemistry profile results were available for analysis and the identified abnormalities are shown in Tables 2 and 3, respectively. On the CBCs, leukopenia characterized by lymphopenia or neutropenia, and a mildly increased hematocrit, were the most common findings. The

most common abnormalities on chemistry profiles included a decreased albumin, decreased total protein, and/or decreased cholesterol.

Serum folate concentrations

Serum folate concentrations were available at three-time points for all dogs that completed the study. In the CE group (Figure 4), no significant difference of serum folate was found between time points for either the oral or the injectable treatment groups. In the EPI group (Figure 5), no significant difference was identified between time points for the oral treatment group, but serum folate concentrations were significantly lower at the second recheck compared to baseline with the injectable supplementation.

Online daily questionnaire for owners

The owners of 19/27 dogs in the CE group (70%, 10 dogs in the oral and 9 dogs in the injectable treatment group) and the owners of 9/19 dogs in the EPI group (47%, 4 dogs in oral and 5 dogs in injectable treatment group) finished the questionnaire for more than three quarters of the total study duration. One possible adverse effect of injectable cobalamin supplementation was identified by the owner of one dog with EPI. The owner identified the development of a new visible rash under the chin, as well as discolored skin and alopecia around the nose. These abnormalities were first noted about two weeks after starting the weekly cobalamin injections. No adverse effects related to oral cobalamin supplementation were observed based on the questionnaire data.

Discussion

When serum MMA concentrations were compared over time only in dogs having MMA concentrations within RI at baseline (13 dogs with CE and 8 dogs with EPI), no significant decrease was identified with either oral or injectable cobalamin supplementation in both CE and EPI group which is different from the previous study showing significant decrease between baseline and the first recheck (day 28).[20] This dissimilar finding between the two studies might not be relevant because there were only small numbers of dogs in each treatment group having MMA concentrations within the RI at baseline in our study as shown in Figure 8 & 9. Interestingly, there were 9 dogs (5 dogs with CE and 4 dogs with EPI) that showed increased serum MMA concentrations after cobalamin supplementation, and 3 of these dogs (2 dogs with CE and 1 dog with EPI) had serum cobalamin concentrations of less than 400 ng/L after either oral or injectable cobalamin supplementation. This increase of MMA concentrations might represent normal fluctuations within the RI or hypocobalaminemia that was unresponsive to cobalamin supplementation.

All of the owners of dogs receiving oral cobalamin supplementation reported the chew as easy to administer to their dogs. Based on the questionnaire results, some dogs with CE or EPI in both treatment groups had intermittent GI signs (vomiting or diarrhea) during the study. Those GI signs might be related to the underlying diseases, different medications to treat the underlying diseases prescribed by their primary veterinarians, or oral cobalamin administration.

Among the excluded 38 dogs, 23 dogs were excluded due to a normalized serum cobalamin concentration at baseline even though they had cobalamin concentrations lower than the lower limit of the RI upon initial evaluation for eligibility. The duration between initial evaluation of eligibility and baseline varied at the most by a few months and serum cobalamin concentrations at baseline of the

excluded dogs was mainly low normal (< 400 ng/L). These findings might indicate a natural fluctuation of serum cobalamin concentration, improvement of the underlying disease process, or unreported cobalamin supplementation by some owners. Unreported cobalamin supplementation by owners could occur in dogs with EPI because a product containing both pancreatic enzymes and cobalamin is available (Panzquin, Nutramax Laboratories). Also, a diet change might be a cause of unreported cobalamin supplementation because a recent study showed that dogs fed with a balanced processed commercial diet had significantly higher cobalamin concentrations in plasma than dogs fed with a commercial raw diet. However, in this study, only one breed (Staffordshire Bull Terriers) was evaluated and cobalamin concentrations were mostly within the RI regardless of the type of diet.[32]
